# 'Just Say No' (Or at Least Ask Why) STOMP Medication Reviews in Tower Hamlets Community Learning Disability Service

**DOI:** 10.1192/bjo.2022.504

**Published:** 2022-06-20

**Authors:** Anthony Jones, Shiva Fouladi-Nashta, Nicole Eady, Simon Bedeau, Hannah Hafezi

**Affiliations:** East London NHS Foundation Trust, London, United Kingdom

## Abstract

**Aims:**

'STOMP stands for stopping over medication of people with a learning disability, autism or both with psychotropic medicines. It is a national project involving many different organizations which are helping to stop the over use of these medicines. STOMP is about helping people to stay well and have a good quality of life'. Our aim was to reduce the percentage of psychotropic burden on the LD and/or autism caseload in Tower Hamlets.

**Methods:**

We reviewed the internal LD caseload that fit STOMP eligibility criteria (prescribed antipsychotics without an indicated mental health diagnosis).

We calculated the% of BNF maximum dose for individual service users, aimed to reduced this, and reviewing the cumulative dose reduction achieved across the service, before and after an intervention.

The primary intervention was the introduction of a pharmacy led clinic for service users meeting the criteria. This allowed closer f/u from LD pharmacist, thorough medication histories independent of their routine psychiatric reviews, and using GASS and BAI scales to quantify change achieved to their quality of life.

We used early and rigourous people participation to consider the role medications (and their overprescription) in service users quality of life, and asked what service users want out of these medication reviews. Several focus groups were ran without People Participation Lead.

**Results:**

Prior to starting of clinic - Of 29 STOMP eligible patients within TH CLDS, we have reduced antipsychotics in 8 of them through general raising awareness of STOMP (presentations to staff, reviews of GP letters to identify service users within the caseload who are likely to benefit and/or be receptive to dose reductions etc). So far total reduction of 45.4%, (and a total of three patients have been stopped all together).

**Conclusion:**

The majority of the results and intervention are yet to be collated, and we are collecting these over the next 2 months, but provisionally we hope to conclude that by reducing the quantity of psychotropic medication we prescribe will improve the quality of life for our service users

